# Deep Learning Based Inter-subject Continuous Decoding of Motor Imagery for Practical Brain-Computer Interfaces

**DOI:** 10.3389/fnins.2020.00918

**Published:** 2020-09-30

**Authors:** Sujit Roy, Anirban Chowdhury, Karl McCreadie, Girijesh Prasad

**Affiliations:** ^1^School of Computing, Engineering & Intelligent Systems, Ulster University, Derry-Londonderry, United Kingdom; ^2^School of Computer Science and Electronic Engineering, University of Essex, Colchester, United Kingdom

**Keywords:** convolutional neural network (CNN), deep learning, motor imagery, brain-computer interface (BCI), electroencephalography (EEG), adaptive learning, SGDM, ADAM

## Abstract

Inter-subject transfer learning is a long-standing problem in brain-computer interfaces (BCIs) and has not yet been fully realized due to high inter-subject variability in the brain signals related to motor imagery (MI). The recent success of deep learning-based algorithms in classifying different brain signals warrants further exploration to determine whether it is feasible for the inter-subject continuous decoding of MI signals to provide contingent neurofeedback which is important for neurorehabilitative BCI designs. In this paper, we have shown how a convolutional neural network (CNN) based deep learning framework can be used for inter-subject continuous decoding of MI related electroencephalographic (EEG) signals using the novel concept of Mega Blocks for adapting the network against inter-subject variabilities. These Mega Blocks have the capacity to repeat a specific architectural block several times such as one or more convolutional layers in a single Mega Block. The parameters of such Mega Blocks can be optimized using Bayesian hyperparameter optimization. The results, obtained on the publicly available BCI competition IV-2b dataset, yields an average inter-subject continuous decoding accuracy of 71.49% (κ = 0.42) and 70.84% (κ = 0.42) for two different training methods such as adaptive moment estimation (Adam) and stochastic gradient descent (SGDM), respectively, in 7 out of 9 subjects. Our results show for the first time that it is feasible to use CNN based architectures for inter-subject continuous decoding with a sufficient level of accuracy for developing calibration-free MI-BCIs for practical purposes.

## 1. Introduction

The practical applications of brain-computer interfaces are often hindered by the need for repeated calibration for each individual participant due to large inter-subject variability in the EEG signal. Even when different sessions on the same participant are considered, BCI systems need recalibration due to the non-stationary nature of the EEG signals leading to inter-session inconsistency (Chowdhury et al., 2018b). BCIs are often used for neurorehabilitation and for developing control and communication systems for patients suffering from various neurological disorders. Often the problem is exacerbated due to the presence of varying brain lesions among users. Studies conducted on patient population alongside healthy individuals have shown such patterns where the variation in BCI performance was more in patient population than in healthy population (Spüler et al., 2012; Chowdhury et al., 2019). With regards to neurorehabilitation especially, the time-consuming calibration process leads to user frustration and a lack of motivation which can hinder the recovery process. This is evident from the work of Morone and colleagues who found a significant correlation between motivation and BCI performance (Morone et al., 2015) which is further found to be strongly correlated with motor recovery (Bundy et al., 2017). General sources of intra- and inter-subject variability leading to the covariate shifts in the dataset include different emotional and mental processes happening in the background of the MI (Saha and Baumert, 2020). Other sources may include the neuroanatomy of the brain for different subjects and the inter-subject difference in the cognitive style of performing a motor-task over time (Seghier and Price, 2018). The volume conduction may also play a major role in covariate shifts in the EEG data (Chowdhury et al., 2018b). Previous attempts to solve this problem involved (1) attempting to discover globally relevant EEG features and (2) the use of adaptive EEG classifiers (Lotte et al., 2018). Recent studies also utilized some BCI performance Predictors to augment the transfer learning process (Saha et al., 2018; Saha et al., 2019).

An extensive detail of transfer learning approaches for BCIs has been given in Jayaram et al. (2016). Transfer learning is often implemented by transferring stationary and/or discriminative information invariant across the subjects (Wang et al., 2015; Gaur et al., 2019a). Apart from globally relevant feature representation, other approaches to transfer learning involve ensemble learning, sparse subset of spatial filters, and classifiers (Fazli et al., 2009; Tu and Sun, 2012; Raza et al., 2019), and domain adaptation of classifiers (Vidaurre et al., 2011). A variant of the popularly used common spatial pattern (CSP) based spatial filtering, called composite CSP, proposed by Kang and colleagues, was one of the earliest efforts of inter-subject transfer learning using EEG signals (Kang et al., 2009). Regularized CSP filters derived from other subjects also gave significant performance improvement for inter-subject transfer learning (Devlaminck et al., 2011; Lotte and Guan, 2011). Another popular method of intra- and inter-subject transfer learning is covariate shift adaptation by combining the unlabeled test data with the labeled training data which corrects the covariate shifts arising from the changes of marginal distribution between different subjects/sessions (Li et al., 2010; Arvaneh et al., 2014). Some different approaches are also proposed for inter-subject transfer learning where event-related cortical sources are estimated from subject independent EEG recordings (Saha et al., 2019) which can compensate for the changes in head morphology and electrode positioning (Wronkiewicz et al., 2015). In a recent study, a Riemannian geometry-based approach is successfully applied for cross-subject and cross-session transfer learning which significantly improved BCI performance (Zanini et al., 2018; Gaur et al., 2019a). Others have also used novel filtering techniques using multivariate empirical mode decomposition (MEMD) along with CSP features for subject independent learning and have shown improved performance on BCI Competition IV-2a dataset (Gaur et al., 2019a,b). Halme and colleagues compared several different methods for cross-subject decoding of MI and passive movements using both EEG and MEG signals. They found better cross-subject accuracy in MEG as compared to EEG for an MI task (70.6%) (Halme and Parkkonen, 2018). Transfer learning was also realized using a covariate shift adaptation technique for session-to-session transfer, although their effect on inter-subject learning is still uncertain (Chowdhury et al., 2018b). Other attempts of suppressing subject-specific calibration include Kalman filter-based decoder (Sussillo et al., 2016) and actor-critic based reinforcement learning (Pohlmeyer et al., 2014; Prins et al., 2017). So far the evidence of high performing inter-subject transfer learning models is scarce and mostly concentrates on event-related potentials (Jin et al., 2013; Kindermans et al., 2014). Of late, the use of a Sparse Group Representation Model showed promising results for inter-subject decoding which compensated reduced recoding from the same subject by making use of previously recorded data from other subjects (Jiao et al., 2019).

Conventional methods of inter-subject transfer learning mentioned above are heavily dependant on feature engineering techniques which limit their capacity to be applied on a large variety of subjects. Recently, following the success of deep learning-based algorithms in image processing applications, inroads have been made in the field of biomedical engineering, especially in the classification of brain signals where reliable and stable performance is still a challenge after more than two decades of research (Roy et al., 2019).

Lu and colleagues proposed a deep belief network method using a restricted Boltzmann machine (RBM) for MI classification (Lu et al., 2017). Different architectures of deep convolutional neural networks (CNNs) have also been explored for decoding EEG signals (Schirrmeister et al., 2017). A CNN with stacked autoencoders (SAE) has been shown to achieve better classification accuracy on BCI competition IV-2b dataset than the traditional classification approaches (Tabar and Halici, 2016; Zubarev et al., 2018; Roy et al., 2019a). Recently, Bayesian extreme learning was also proposed for improving the performance of MI-BCIs (Jin et al., 2020). However, none of these deep learning-based decoders addressed the issue of inter-subject transfer learning in BCI, except for some recent studies (Lawhern et al., 2018; Fahimi et al., 2019; Kwon et al., 2019). Even in these studies, the issue of continuous feedback was not addressed while it is of utmost importance that a BCI, especially for neurorehabilitation applications, should be capable of providing continuous neurofeedback contingent to task-dependent neural activity. The paper therefore proposes the novel concept of Mega Blocks for adapting a CNN architecture to tackle inter-subject variabilities, and validates for the first time the feasibility of such a CNN-based architecture for inter-subject continuous decoding of MI-related EEG signals. The study is important as it paves the way for calibration-free BCI designs based on CNN which can be used for vital practical purposes such as providing neurofeedback in a rehabilitative BCI setting reducing the user frustration related to the need to recalibrate. Another important aspect of this study is that it utilizes publicly available data for the validation which means that the work can be reproducible and serve as a benchmark for further development in a similar direction. The results of intra-subject and single-trial classification accuracies using the same CNN architectures are also provided for the sake of comparability.

## 2. Materials and Methods

### 2.1. Dataset

**Figure 1:**
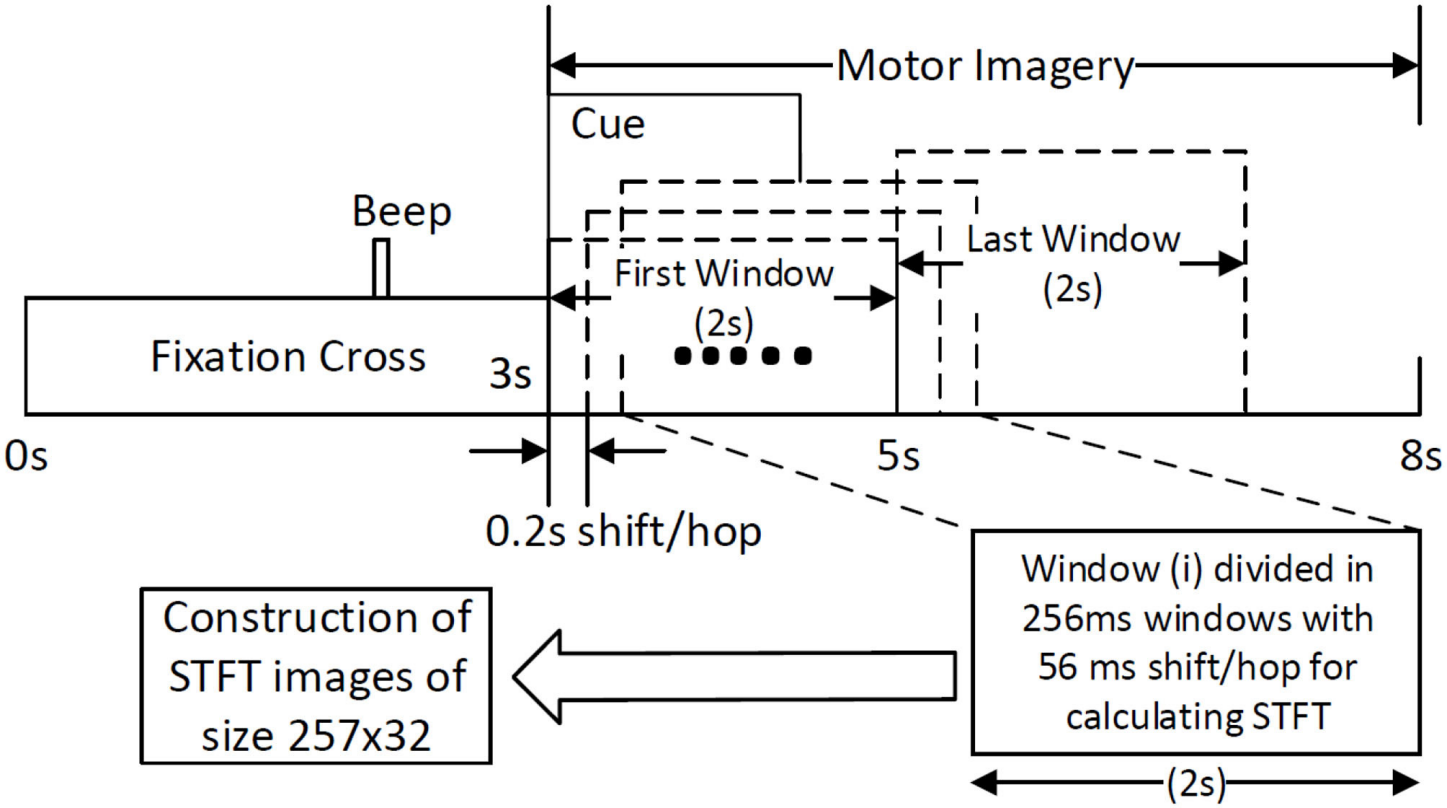
Construction of STFT images by sliding window of size 2 s with a shift/hop of 200 ms is divided into 256 ms sub-windows (with 56 ms shift/hop) for calculating STFT of the MI period within the trial.

BCI competition IV-2b is a well-known dataset and is used as a benchmark for testing new algorithms in the area of MI-based BCI (BCI-Competition, 2008). The dataset comprises of EEG data recorded from 9 healthy participants. The data were recorded in 5 sessions, where the first 3 sessions are for calibrating an EEG decoder and the last 2 sessions are for evaluation purposes. Three channels on the primary motor cortex, C3, Cz, and C4 were used for the bipolar recording of EEG signals at the sampling rate of 250 Hz. Signals were band-passed between 0.1 and 100 Hz with a notch filter at 50 Hz set at the time of recording using signal acquisition hardware. Each session consists of equally distributed trials of left and right hand MI classes. The timing diagram (Figure 1) shows that each trial started with a fixation cross for 3 s, after which a cue appears as an arrow for 1.5 s instructing the participant to do left or right-hand MI. After the MI period of 4 s, there was a short break of a few seconds until the start of the next trial. The only difference between the trials at the calibration and evaluation phase is that for the evaluation phase a happy or sad smiley was shown during the MI period as feedback. In our study, we have trained the CNN classifier on the trials of the first 3 sessions' data (total 420 trials) and tested on the last 2 sessions' data (total 320 trials).

### 2.2. Input Image Construction

**Figure 2:**
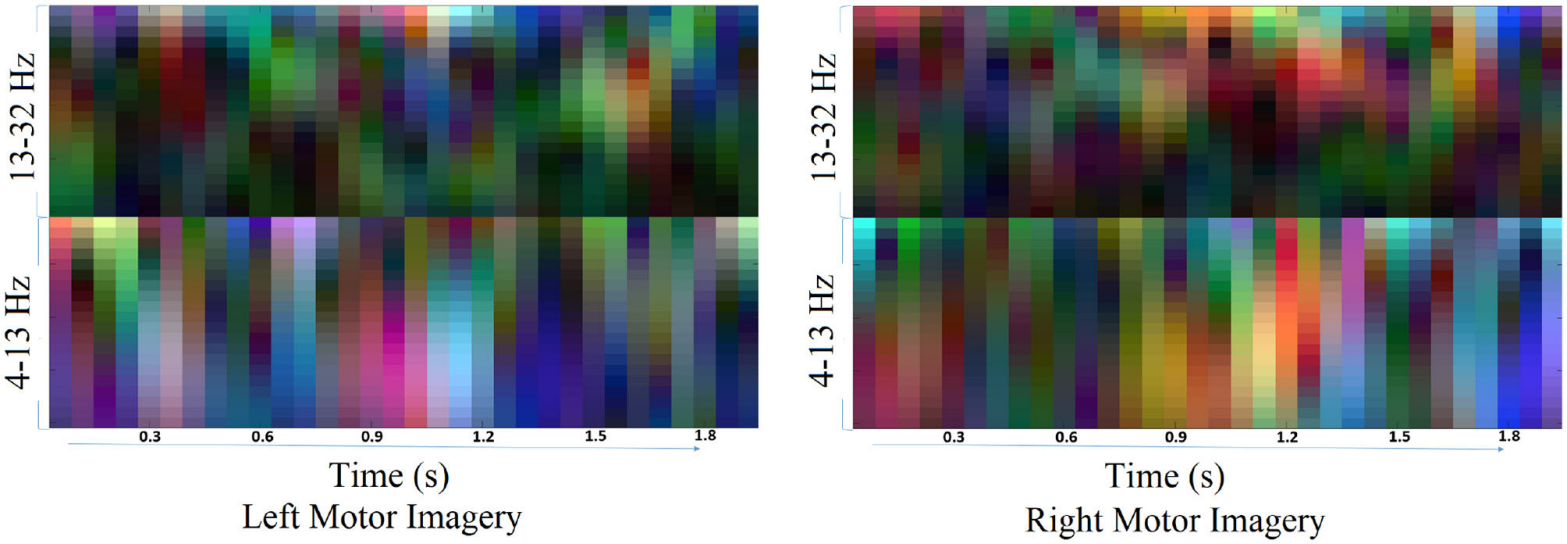
Example of input images for left and right MI. The input images are formed by combining the theta-alpha and beta band STFT images vertically. The three EEG channels: C3, Cz, and C4, are stacked depthwise, the same as an RGB image. These images are then fed into the CNN for classification purposes.

The traditional approach of classifying EEG signals is based on extracting time-frequency based features and training using traditional classifiers such as linear discriminant analysis (LDA), or support-vector-machine (SVM) (Chowdhury et al., 2018a). A CNN typically takes the input as an image; it is well-known that vital information is contained within the time-frequency spectrogram of EEG signals popularly known as event-related desynchronization/synchronization (ERD/ERS) in the context of MI (Chowdhury et al., 2019). Hence, a similar approach was followed for constructing input images for CNN, wherein short time Fourier transform (STFT) was used for obtaining the time-frequency spectra of the MI related changes in the EEG signal. The STFT is evaluated on a time period of 2 s within the MI period of a trial (i.e., between 3 and 7 s), which is shifted by 200 ms, thereby generating 11 input images per trial. In our previous study on the clinical effect of BCI based continuous anthropomorphic multimodal neurofeedback on stroke patients (Chowdhury et al., 2018a), the shift between the two consecutive windows was set as 500 ms which was sufficient but suffered from high latency. In order to reduce the latency by making it closer to real-time, in the present study we decreased the shift by 300 ms to set it as 200 ms. Although some studies used latencies as low as 72 ms (Foldes et al., 2015), we made a trade-off between the amount of overlap and latency to avoid reducing it further. The choice of the time window motivated by the fact that, as we have considered frequencies as low as 4 Hz, i.e., time period of 250 ms, we kept the size of the time window sufficiently high (i.e., 2,000 ms) to allow 8 oscillations of the lowest frequency for proper bandpass filtering. Thus the combination of a 2 s time window and 200 ms shift makes 11 segments within the 5 s MI period producing 11 images in a single trial. This design would be useful when it comes to providing continuous neurofeedback in a more intuitive way and for which having low latency is an essential criterion (Foldes et al., 2015). But unlike these previous studies (Foldes et al., 2015; Chowdhury et al., 2018a) which are primarily based on within-subject learning, we have shown how continuous feedback could be incorporated into a CNN based inter-subject transfer learning setting which can then contribute to calibration-free neurorehabilitative BCI designs without compromising the richness of the neurofeedback. As the sampling frequency of the EEG signal is 250 Hz, a 2 s signal is composed of 500 samples. We have chosen a window size of 64 samples, with an overlap of 50 samples between the consecutive windows. The number of fast-Fourier-transform (FFT) points was 512. Thus the size of the spectrogram was 257 × 32, where 257 was the number of frequency components and 32 was the number of time points. Event related desynchronization (ERD) and event-related synchronization (ERS) phenomena typically occur over the frequency ranges 8–13 and 13–32 Hz, respectively (Pfurtscheller and da Silva, 1999). In one of the earlier works on CNN based MI-BCI, Tabar and Halici (2016) have used the 6–13 Hz frequency band for STFT plots with satisfactory accuracy. This shows a partial inclusion of theta band (4–7Hz) along with the alpha (8–13 Hz) band for generating STFT plots. Hence, in our approach, we have combined the entire theta and alpha band (4–13 Hz) along with the beta band (13–32 Hz) to capture all possible neurodynamics related to the MI. From this spectrogram, we first choose the theta-alpha-spectrogram for 4–13 Hz which was of the size 20 × 32. Then we choose beta-spectrogram for 13–32 Hz, which was of size 41 × 32. To match the sizes of these two sub-spectrograms (by sub-spectrograms we mean the theta-alpha-spectrogram and beta-spectrogram as they are the subsets of the initial spectrogram of size 257 × 32 after STFT) we used cubic interpolation on the beta-spectrogram and reduced it to size 20 × 32 so that the effect of both the bands remained the same on the final input to the CNN. A similar approach can also be found in Tabar and Halici (2016) where the same cubic interpolation was applied to match the sizes of the two spectrograms. The theta-alpha-spectrogram and beta-spectrogram are concatenated vertically to get a spectrogram of size 40 × 32. Thus the spectrograms of size 40 × 32 are calculated for each of the three EEG channels C3, Cz, and C4. The final image is constructed by concatenating these three spectrograms on a third dimension orthogonal to the time-frequency plane. So, the size of the final image becomes 40 × 32 × 3, where *N*_*f*_ = 40, *N*_*t*_ = 32, and *N*_*ch*_ = 3. This construction process of the STFT images is shown in Figure 1. An example of input images formed out of the STFT images, for left and right-hand MI is shown in Figure 2. The frequency ranges stacked on top of each other are the 4–13 Hz range (combining the theta and alpha bands) and the 13–32 Hz range (the beta band). The colors in Figure 2 represents the mixed intensity of three EEG channels C3, Cz, and C4 which are stacked depthwise similar to RGB images. These input images are then decoded by the CNN for generating the neurofeedback.

### 2.3. Architecture-1 for Intra-Subject Learning

The Architecture-1 is defined with 16 filters of size 3 × 3 with a stride of 1 for the first convolutional layer. An input image of 40 × 32 × 3 was used as an input to this convolutional layer. After the first convolutional layer, batch normalization and maxpooling were performed using a filter of 3 × 3 and a stride of 2. Again, for the next convolutional layer, 32 filters were used of size 3 × 3 and similarly, maxpooling was performed with a factor of 3 and a stride 2. After that, another convolutional layer was added with 64 filters of 3 × 3 size and a stride of 1. Finally, a fully connected layer average pooling was performed with a factor of 8 and a stride of 1. For learning the parameters of the CNN two different training methods used are stochastic gradient descent method (SGDM) and adaptive moment estimation (Adam).

The *k*-th feature map at a given layer can be represented as:

(1) $$\begin{array}{l} h_{ij}^{k}=f\left(a\right)=f\left({\left(w^{k}\times x\right)}_{ij}+b_{k}\right) \end{array}$$

where *x* is the input image, *w*^*k*^ is the weight matrix and *b*_*k*_ is the bias value for *k* = (1, 2, ..., 30). The output function *f* is selected as rectified linear unit (*ReLu*) function and it is approximated as *softplus* function defined as,

(2) $$\begin{array}{l} f_{a}=ReLU\left(a\right)=ln\left(1+e^{a}\right) \end{array}$$

The Gradient descent method attempts to minimize an objective function *J*(θ) which is parameterized by a model's parameter (where θ ∈ ℝ^*d*^) by updating the parameters in the steepest descent direction from the gradient of the objective function ∇_θ_*J*(θ). The learning rate is defined by the size of steps considered to reach a local minimum. However, at each step, gradient descent requires evaluation of n derivatives, which is expensive. A popular modification is *SGD* (Johnson and Zhang, 2013), where at each iteration (*t* = 1, 2,.) *w*^*t*^ is defined as follows:

(3) $$\begin{array}{l} w^{t}=w^{\left(t-1\right)}-\eta \nabla \psi \left(w^{\left(t-1\right)}\right) \end{array}$$

where η is the learning rate and ψ represents the loss function. In a simpler way, learning of the model parameters can be expressed as Equation (4), where parameters perform an update for each training example *x*^(*i*)^ and label *y*^(*i*)^.

(4) $$\begin{array}{l} \theta =\theta -\eta .\nabla_{\theta }J\left(\theta ;x^{\left(i\right)};y^{\left(i\right)}\right) \end{array}$$

The advantage of SGDM is that computation time is 1/n of standard gradient descent as every step depends upon a single derivative ∇ψ_*i*_(·). The Momentum (Qian, 1999) method helps SGD to accelerate in applicable direction by damping oscillations through the addition of the fraction μ of the update vector to the current update vector. As shown in Equation (5), μ can be considered as a momentum decay coefficient where μ ∈ [0, 1), which controls the rate at which old gradients are discarded.

(5) $$\begin{array}{l} v_{t+1}=\mu .v_{t}-\eta .\nabla l\left(\theta \right) \end{array}$$

(6) $$\begin{array}{l} \theta_{t+1}=\theta_{t}+v_{t+1} \end{array}$$

**Figure 3:**
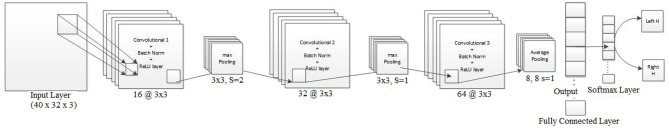
Visualization of Architecture-1 for intra-subject classification. Lines represent the connection between the feature maps. The network starts with 3D input to the convolution which is represented as (image height, image width, and number of channels), i.e., 40 × 32 × 3, and afterward 2D convolution is performed with parameters shown in Table 1.

Architecture-1 has a convolutional 2D layer with *l*2 regularization of 0.0014 and *ReLU*-activation. The details of the parameters are shown in Table 1. Batch normalization was done and the model was trained for 55 epochs with a batch size of 40. For validation, 500 samples were randomly used. The learning rate for the model was 6.7929e^−04^ and the initial momentum was 0.9799. The dropout rate was 0.1 and the drop period was 20. The loss function was cross-entropy which was expressed as Loss = $\overset{N}{\underset{i=1}{\sum }}\overset{K}{\underset{j=1}{\sum }}t_{ij}\ln\;y_{ij}$. The hyperparameters are chosen using Bayesian optimization. Apart from SGDM we have also used Adam as an optimizer on the same CNN architecture (Architecture-1) for tuning the hyperparameters. This is because for some participants (participants 2 and 3) the data were particularly noisy which made the convergence of SGDM very slow. Hence, the experimentation was also done using Adam as an optimizer for faster convergence using a large learning rate. It is to be noted that aside from the change in the optimizer (i.e., from SGDM to Adam) the layers of the CNN Architecture-1 were exactly the same as described in Table 1. The corresponding architecture diagram is shown in Figure 3.

**Table 1:** Parameters for Architecture-1 for intra-subject learning.

**Layers**	**Filters**	**Size**	**Options**
**Descriptions of the design parameters for Architecture-1**			
Image input		[40, 32, 3]	
layers			
Convolution	16	[3, 3]	Stride = [1,1]
2D layer			
Batch norm			10^−5^
ReLU layer			
Maxpooling 2D		[3, 3]	Stride = [2,2]
layer			
Convolution	32	[3, 3]	Stride = [1, 1]
2D layer			
Batch norm			10^−5^
ReLU layer			
Maxpooling 2D		[3, 3]	Stride = [2, 2]
layer			
Convolution	64	[3, 3]	Stride = [1, 1]
layer			
Batch norm			10^−5^
ReLU layer			
Average pooling		[8, 8]	Stride = [1, 1]
layer			
Fully connected		192	
layerlayer Softmax layer			
Classification			Loss =
output layer			cross entropyex

Adam can be understood as a combination of SGDM with momentum and Root Mean Square Error Propagation (*RMSprop*). It is an adaptive learning rate method, where the learning rate is computed from different parameters. Adam keeps exponentially decaying the average of past gradients *mt* similarly to momentum.

Adam uses an exponentially moving average which is computed on the current mini-batch gradient:

(7) $$\begin{array}{l} m_{t}=\beta_{1}m_{t-1}+\left(1-\beta_{1}\right)g_{t} \end{array}$$

(8) $$\begin{array}{l} v_{t}=\beta_{2}v_{t-1}+\left(1-\beta_{2}\right)g_{t}^{2} \end{array}$$

where *m*_*t*_ and *v*_*t*_ are an estimation of the mean and uncentered variance of gradient (*g*) and β_1_ and β_2_ are new hyperparameters.

The update rule for Adam is

(9) $$\begin{array}{l} \theta_{t+1}=\theta_{t}-\frac{\eta }{\sqrt{{\hat{v}}_{t}}+\varepsilon }{\hat{m}}_{t} \end{array}$$

where θ is the model parameter, θ ∈ ℝ^*d*^, and η is the learning rate.

The proposed default values are 0.9 for β_1_, 0.999 for β_2_, and 10^−8^ for ϵ (Kingma and Ba, 2014). It was shown empirically that Adam is effective in practice and quite popular as compared to other adaptive learning-method algorithms. For Adam, the initial learning rate was 0.01 using a batch size of 50 and the model was trained for 15 epochs.

The Bayesian optimization method was used for selecting the best hyperparameters for the model. The range of parameters for the convolutional layer was set from 1 to 5, the learning rate ranged from *e*^−06^ to *e*^−02^, the momentum in the case of SGDM was from 0.6 to 0.98, and the L2 regularization was from *e*^−10^ to *e*^−02^ for a total of 30 different objective functions to evaluate. The Bayesian optimization method tries to minimize the scalar objective function *f*(*x*) for *x* in a bounded set. The deterministic or stochastic function can obtain similar/different results for evaluation of the same point *x*. There are several steps to minimize, which include Gaussian process model of *f*(*x*), and acquisition function *a*(*x*) based on the model of *f*(*x*) which is maximized for the next point *x* for evaluation. The acquisition functions evaluate the “goodness” of a point *x* based on the posterior distribution function *Q* (Gelbart et al., 2014). Bayesian optimization estimates the smallest feasible mean of posterior distribution by sampling several thousand points within variable bounds and improving them using local search.

Expected improvement (*EI*) of acquisition function evaluates the acquisition function, ignoring values responsible for the increase in the objective. *EI* can be expressed as:

(10) $$EI(x,Q)=E_{Q}[max(0,\mu_{Q}(x_{b})-f(x)]$$

where *x*_*b*_ is the location of the lowest posterior mean and μ_*Q*_(*x*_*b*_) is the lowest value of posterior mean.

The Probability of improvement (*PI*) optimization function calculates the probability of a better objective function value by a new point *x* which is modified by a margin parameter *m*. *PI* is given as,

(11) $$\begin{array}{l} PI\left(x,Q\right)=P_{Q}\left(f\left(x\right)<\mu_{Q}\left(x_{b}\right)-m\right) \end{array}$$

where *m* is considered as the estimated noise standard deviation and the probability is evaluated as,

(12) $$\begin{array}{l} PI=\Phi \left(v_{Q}\left(x\right)\right) \end{array}$$

Here Φ(.) is the unit normal Cumulative Density Function and

(13) $$\begin{array}{l} v_{Q}\left(x\right)=\frac{\mu_{Q}\left(x_{best}\right)-m-\mu_{Q}\left(x\right)}{\sigma_{Q}\left(x\right)} \end{array}$$

where σ_*Q*_ is the posterior standard deviation of the Gaussian process at *x*.

### 2.4. Architecture-2 for Inter-subject Transfer Learning

In the case of transfer learning, the dataset was huge as the classifier needed to learn from all 8 subjects over 5 sessions. Since we have a mixed dataset it was important to account for variability over the sessions and over subjects. For performing transfer learning, huge networks are often used such as ResNet50 (He et al., 2016), AlexNet (Krizhevsky et al., 2012) in the case of image classification. But in the domain of BCI, data collection is a slow process and hence limited in size. Therefore, we needed to design an adaptive system to account for the noise and non-stationarity arising across various sessions and subjects. Thus, we designed Mega Blocks which has the capacity to repeat the specific architecture block over time. For example, in one Mega Block, we can put one or more convolution layers, the parameters of which are exactly the same as the corresponding Mega Block in the number of filters, filter size, activation function, and L2 regularization. The fixed parameters can be replicated for every convolution block inside Mega Block, which ranges from 1 to 5 in our case, and can be extended further. It is advised to add one or more Mega Blocks instead of adding more than 5 convolutional layers inside a Mega Block as the addition of more convolutional layers inside a Mega Block will increase the training parameters significantly. Also, the addition of more Mega Blocks will help in learning more micro-features. After every Mega Block, there can be maxpooling/averagepooling layer whose output is given as the input to the next Mega Block. The parameters of a Mega Block are optimized using Bayesian hyperparameter optimization, which includes, the number of convolution layers, learning rate, momentum, and regularization. Using this methodology we have observed that the trained model is less vulnerable to noisy subjects' data considering the amount of good data is significantly higher. The model can be further modified by introducing skip layers much like ResNet50 inside Mega Blocks. One Mega Block can extend itself from 1 convolution block to 5 convolution blocks with similar properties. Each convolution block has a convolutional layer connected with a batch normalization layer and a *ReLU* layer. After every Mega Block, a *maxpooling* layer was added whose output was fed to the next Mega Block input. Finally, the average pooling layer is connected with a fully connected layer, *softmax* layer and classification. The loss for training was set to cross-entropy. The design parameters of Architecture-2 are shown in Table 2.

**Table 2:** Design Parameters for Architecture-2 for inter-subject transfer learning.

**Layers**	**Filters**	**Size**	**Activation**	**Options**
Image input layer		[40, 32, 3]		
Mega Block 1	9	[5,5]	relu	Stride = [1,1] bnm = 10^−^5
Maxpooling layer		[3,3]		Stride = [2,2]
Mega Block 2	18	[3,3]	relu	Stride = [1,1] bnm = 10^−^5
Maxpooling layer		[3,3]		Stride = [2,2]
Mega Block 3	36	[3,3]	relu	Stride = [1,1] bnm = 10^−^5
Average pooling layer		[8,8]		Stride = [1,1]
Fully connected layer		108		
Softmax layer				
Classification output layer			2	Loss = cross entropyex

**Figure 4:**
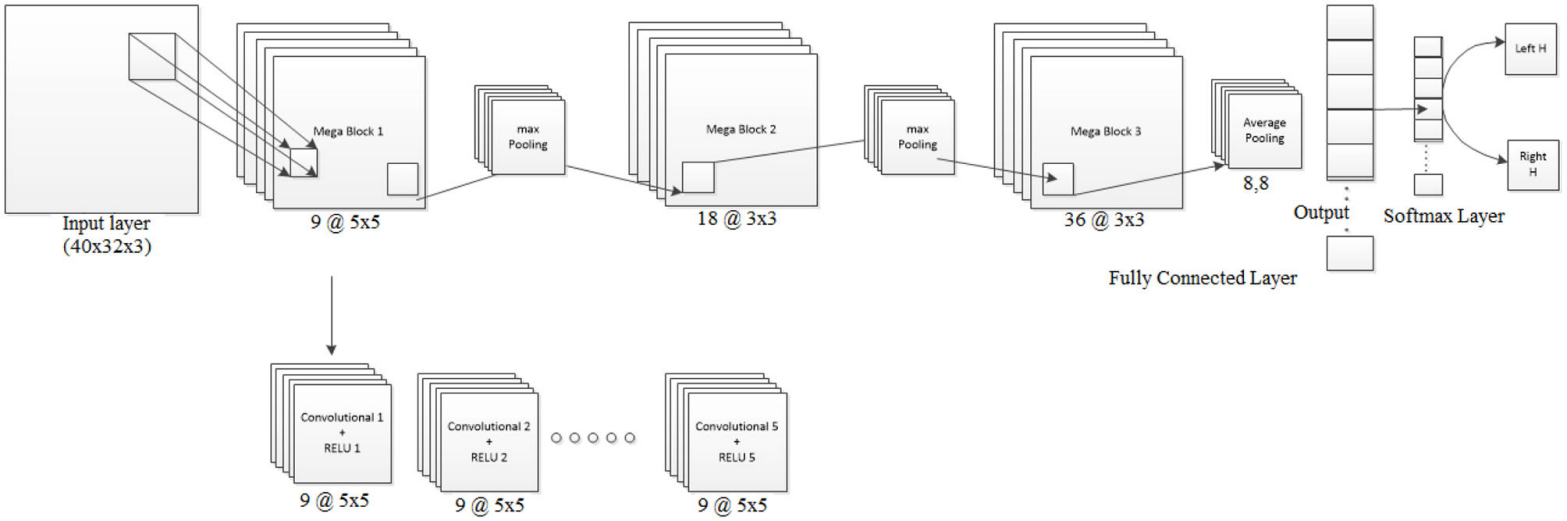
Visualization of Architecture-2 for inter-subject classification. Lines represent the connection between the feature maps. The Mega Block is the combination of 5 convolution blocks of the same size and number of filters. The number of convolution blocks required is learned through Bayesian optimization. The network can extend itself from 3 convolution layers to 15 or more convolution layers, depending on the amount of data and noise level. The optimization works by defining the objective function and finding its minimum observed value. The network designed is useful for representing the input data in small dimensional representation to avoid noise.

It is to be noted that similar to Architecture-1, Architecture-2 was also trained using both the optimizers: SGDM and Adam. Table 3 shows the number of convolution blocks used inside Mega Blocks 1, 2, and 3 in the case of training methods as SGDM and Adam. The number of maximum epochs for training was 50 and mini-batch size was 64. The learning rate drop rate factor was 0.1 and the drop period was 40. A general overview of the CNN architecture used here is shown in Figure 4 which evolves into Architecture-1 (for intra-subject learning) or Architecture-2 (for inter-subject learning) depending upon the choice of parameters given in Tables 1, 2, respectively.

**Table 3:** Number of convolutional layers for each subject used in Architecture-2 for inter-subject transfer learning purpose (MB = Maga Block).

**Subjects**	**No. of conv. blocks (SGDM)**			**No. of conv. blocks (Adam)**		
	**MB 1**	**MB 2**	**MB 3**	**MB 1**	**MB 2**	**MB 3**
S01	1	1	1	1	1	1
S02	3	3	3	1	1	1
S03	1	1	1	1	1	1
S04	3	3	3	5	5	5
S05	3	3	3	3	3	3
S06	1	1	1	1	1	1
S07	1	1	1	1	1	1
S08	2	2	2	4	4	4
S09	1	1	1	1	1	1

### 2.5. Training and Continuous Decoding

CNN was evaluated for continuous decoding of MI meaning that rather than making a decoding once within a trial, we are decoding multiple times. To facilitate this we divided the trial into multiple windows of size 2 s, which were shifted by 200 ms (i.e., 1,800 ms of overlap). Thus every trial was divided into 11 segments and the decoding was done by the CNN based classifier for each of the segments. To keep parity in the signal processing of the training and feedback stages, similar segmentation was also performed for training data also. All the 11 segments of a particular training trial were assigned the same class-label while feeding into the CNN. One advantage of such segmentation is that we can increase the training instances for CNN, as we know that the deep learning classifiers require a larger training data set. Thus rather than having 420 training examples for 420 trials, we had 420 × 11 = 4,620 training examples. In this way, the CNN classifier can generate decodings every 200 ms interval within a trial and can provide continuous feedback to the participant accordingly.

The performance of the CNN architectures is evaluated by calculating the classification accuracies in three different manners, gross classification accuracy (*CA*_*gross*_), single-trial classification accuracy (*CA*_*ST*_), and optimal time-point classification accuracy (*CA*_*opt*_). The *CA*_*gross*_ is defined as the percentage of correctly classified feedback instances among all the available feedback instances (i.e., 320 × 11 = 3,520, the number of all feedback instances, where 320 is the number of feedback trials across two sessions and 11 is the number of segments into which a single-trial was divided). Next, *CA*_*ST*_ is calculated as follows. To consider a single-trial to be classified correctly, we counted how many segments out of the 11 segments of a single-trial were classified correctly. If the number is 6 or more (i.e., half of the total number of segments are correct) then the feedback trial is considered to be classified correctly. Following this rule, *CA*_*ST*_ is defined as the percentage of correctly classified feedback trials among all the available feedback trials.The rationale behind the choice of such a *CA*_*ST*_ calculation lies in the fact that here we have compared the accuracies of continuous decoding (*CA*_*gross*_) with the single-trial decoding (*CA*_*ST*_) and this comparison would be inconsistent if we define two different time windows for calculating *CA*_*gross*_ and *CA*_*ST*_. Therefore, we needed to come up with a solution for calculating the *CA*_*ST*_ while making use of the same segmentation as in the case of continuous decoding. It is to be noted that the *CA*_*ST*_ and *CA*_*gross*_ measurements are designed in such a way so that they should not be a redundant evaluation of performance. This is because *CA*_*gross*_ deals with all the segments from all trials and does not consider an individual trial separately, which means that it is trial agnostic. On the other hand, *CA*_*ST*_ weighs how many segments (out of 11) within a trial decoded to be a particular class in majority and thereby takes the decision as to how to label that trial. So, it does not consider how many segments across all the trials are classified but how the individual trials are classified. Finally, for *CA*_*opt*_ we have considered only the time segment of maximum accuracy out of the 11-time segments. This means we calculated the accuracy taking one time-segment at a time and assigned the maximum as *CA*_*opt*_ for a particular participant. The reason we presented the performance of the inter-subject transfer learning based on three different accuracy measures *CA*_*gross*_ (for continuous neurofeedback), *CA*_*ST*_ (for single-trial neurofeedback), and *CA*_*opt*_ (for neurofeedback at optimum time point) is that we wanted to validate its feasibility across different BCI paradigms. Some BCI paradigms use continuous neurofeedback, for example in hand rehabilitation where a gradual change in grasp aperture is used (Chowdhury et al., 2018a). To the best of the authors' knowledge, an inter-subject continuous feedback approach based on CNN based transfer learning using the novel concept of Mega Blocks is presented for the first time in this paper. Moreover, it is also worth mentioning that the proposed methodology is also feasible for real-time decoding as the time required for calculation of STFT, image construction, and classification requires approximately 9.32 ms. The optimum time-point for single trial-based decoding is also calculated so that the proposed methodology can be feasible for triggered feedback (Chowdhury et al., 2018b; Chowdhury et al., 2019). The number of trainable parameters in Architecture-1 is 23,269 and for Architecture-2 is between 7,578 and 40,914 depending on the number of convolutional layers inside a Mega Block, which are much smaller than the DeepConvNet architecture (trainable parameters = 152,219,104) (Schirrmeister et al., 2017), Subject-Independent CNN (Kwon et al., 2019) (trainable parameters = 72,264,076) and comparable to the ShallowConvNet architecture (trainable parameters = 40,644) (Schirrmeister et al., 2017). The training time for Architecture-1 (intra-subject) is 794 s which is less than (Tabar and Halici, 2016) where the training time is 1,157 s. The training time for Architecture-2 (inter-subject) is 1934 s which is also less than other inter-subject architecture such as Kwon et al. (2019) where the training time is 12 min. The single-trial decoding time in Tabar and Halici (2016) was 400 ms and in Kwon et al. (2019) it was 150 ms, whereas in the current study the single-trial decoding time is 102.52 ms which is much smaller than others. Thus, it shows that the computational complexity of the proposed CNN architectures is less or comparable to other competitive architectures given in previous studies. It is to be noted that for intra-subject classification the classifier was trained on session 1, 2, and 3 and tested on session 4 and 5 for individual subjects. Additionally, while calculating the accuracy for a particular subject in inter-subject transfer learning case, we have trained the CNN using the session 1 to 5 data from the rest of the subjects. For example, CNN for subject 1 is trained using the data from subject 2 to subject 9. The chance level of these binary classification problems is 50% as there are equal numbers of left and right hand MI trials.

## 3. Results

The performance of the deep learning-based architecture for mental task decoding using EEG is evaluated by calculating the accuracy and the kappa value both for intra- and inter-subject settings. As mentioned in section 2.5, the classification accuracies are calculated in three categories *CA*_*gross*_, *CA*_*ST*_, and *CA*_*opt*_, the results are also presented separately for each one of these. The *CA*_*gross*_ for intra-subject learning is shown in Table 4. The average *CA*_*gross*_ across the trial for Adam was 72.63% ± 13.35. The maximum *CA*_*gross*_ was observed for participant 4 (90.82%), while the minimum observed was 51.88% for participant 3. Indeed, 5 out of 9 participants crossed the BCI performance threshold of 70% (Blankertz and Vidaurre, 2009) in this case. The performance of SGDM for this category resulted in an average classification accuracy of 73.13% ± 14.82, while the maximum accuracy was observed for participant 4 (91.14%) and the minimum observed for participant 3 (54.61%). There was no statistically significant difference (Wilcoxon signed-rank test) between Adam and SGDM performance (*CA*_*gross*_) for intra-subject learning.

**Table 4:** Performance of intra-subject learning for continuous decoding.

**ID**	***CA*_*gross*_**			
	**Adam**		**SGDM**	
	**Test** **(%)**	**Kappa** **(Test)**	**Test** **(%)**	**Kappa** **(Test)**
1	67.57	0.35	68.31	0.37
2	55.83	0.12	55.10	0.10
3	51.88	0.04	54.61	0.09
4	90.82	0.82	91.14	0.82
5	80.65	0.61	80.17	0.60
6	71.78	0.44	72.23	0.44
7	68.28	0.37	67.79	0.36
8	87.77	0.76	88.65	0.77
9	79.12	0.58	80.23	0.60
Mean	72.63	0.45	73.13	0.46
Std	13.35	0.27	14.82	0.26

**Figure 5:**
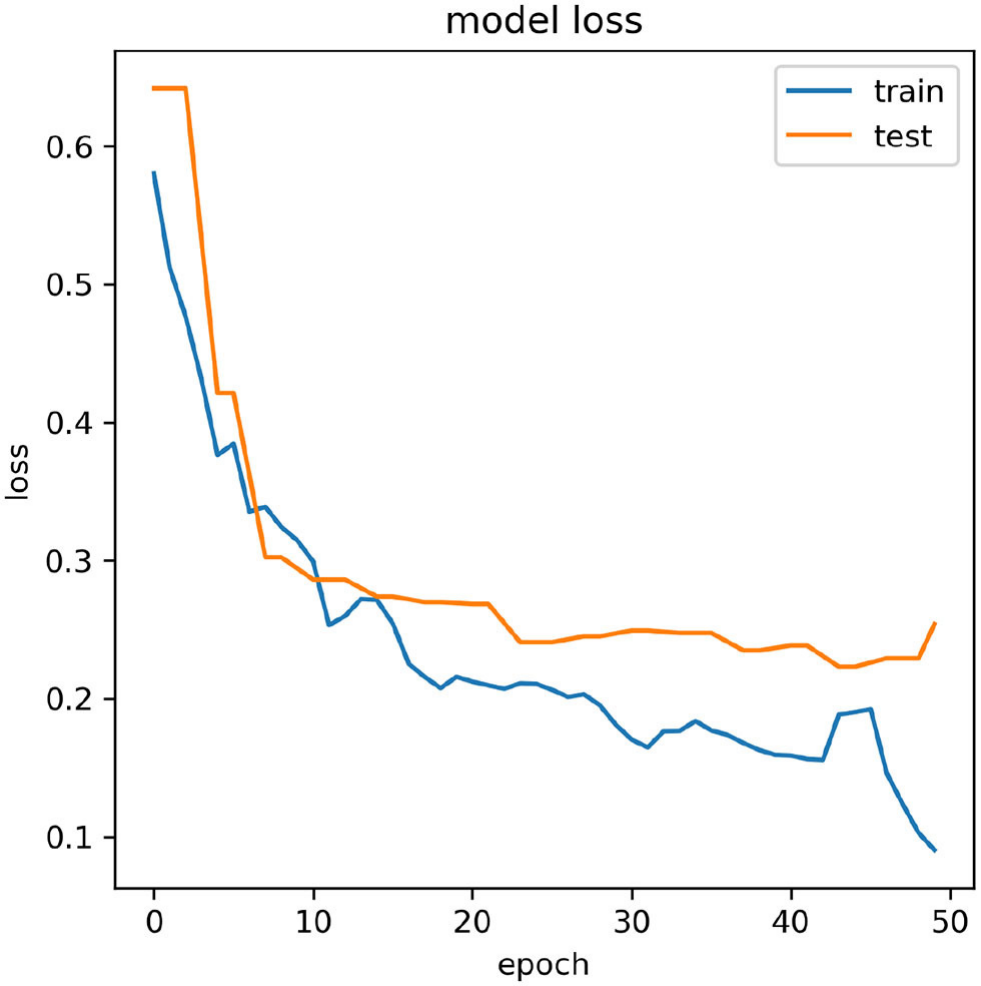
Variation of model loss with the number of epochs for participant 4.

The *CA*_*gross*_ for inter-subject transfer learning is shown in Table 5. Interestingly, although the average *CA*_*gross*_ in the case of Adam (67.78% ± 8.60) is only slightly higher than average *CA*_*gross*_ in the case of SGDM (67.15% ± 8.62), the difference between these two methods (Adam and SGDM) was statistically significant (*p* < 0.05, Wilcoxon signed-rank test). The maximum *CA*_*gross*_ for inter-subject learning was observed for participant 4 for both the methods: 81.42% for Adam and 80.75% for SGDM. The minimum *CA*_*gross*_ was 54.20% in Adam and 54.15% in SGDM; both for participant 3. It is to be noted that the number of participants crossing the BCI performance threshold (70%) for inter-subject learning is 4 in Adam and 3 in SGDM, which is less than what is observed for intra-subject learning. Figure 5 displays the loss vs. epoch for the participant 4. To analyse number of epochs and learning rate for all the participants, data of participant 4, session 1, 2, and 3 were trained, keeping 33% of the cumulative data as validation set. The plot clearly shows that the model does not overfit or underfit as the test errors are converging. For this specific subject a divergence of validation loss be seen at nearly 50 epochs. However, the plot clearly indicates fluctuation which may be due to the low amount of data to train and validate. It is noteworthy that overfitting preventive measures such as batch normalization and dropouts are duly taken while designing the CNN architectures as described in sections 2.3 and 2.4.

**Table 5:** Performance of inter-subject learning for continuous decoding.

**ID**	***CA*_*gross*_**			
	**Adam**		**SGDM**	
	**Test** **(%)**	**Kappa** **(Test)**	**Test** **(%)**	**Kappa** **(Test)**
1	68.23	0.36	68.02	0.36
2	55.41	0.10	54.33	0.09
3	54.20	0.08	54.15	0.08
4	81.42	0.64	80.75	0.61
5	65.40	0.28	64.62	0.29
6	71.59	0.38	68.88	0.38
7	68.53	0.37	67.98	0.36
8	72.13	0.48	72.50	0.45
9	73.13	0.47	73.14	0.46
Mean	67.78	0.35	67.15	0.34
Std	8.60	0.18	8.62	0.17

The performance of the intra- and inter-subject learning for *CA*_*ST*_ is shown in Tables 6, 7, respectively. The average *CA*_*ST*_ and kappa in SGDM for intra-subject learning are found to be 77.31% ± 14.90 and 0.55 ± 0.30, respectively. The maximum performance using SGDM was observed in subject 4 (*CA*_*ST*_ = 95.60%, κ = 0.91), while the minimum was observed in subject 2 (*CA*_*ST*_ = 54.15%, κ = 0.08). In this case, 7 out of 9 participants qualified for the BCI literacy threshold. The average *CA*_*ST*_ and kappa for inter-subject learning with Adam was 77.46% ± 14.52 and 0.55 ± 0.29, respectively. The best and worst performance for Adam in inter-subject learning was found in participant 4 (*CA*_*ST*_ = 94.65%, κ = 0.89) and participant 3 (*CA*_*ST*_ = 54.42%, κ = 0.09), respectively. The BCI literacy threshold was crossed by 7 out of 9 participants in this case. Inter-subject transfer learning performance on the basis of *CA*_*ST*_ resulted in an average accuracy of 70.94% ± 9.89 with kappa 0.42 ± 0.20 for Adam and the average 70.22% ± 9.45 with kappa 0.40 ± 0.19 for SGDM. The maximum accuracy occurred in the case of participant 4 in both the methods with 86.26% (κ = 0.73) for Adam and 83.95% (κ = 0.68) for SGDM. There was no statistically significant difference (Wilcoxon signed-rank test) between Adam and SGDM on the basis of *CA*_*ST*_ and in both cases, 6 out of 9 participants qualified for the BCI literacy threshold.

**Table 6:** Performance of intra-subject learning for single-trial decoding.

**ID**	***CA*_*ST*_**			
	**Adam**		**SGDM**	
	**Test** **(%)**	**Kappa** **(Test)**	**Test** **(%)**	**Kappa** **(Test)**
1	74.92	0.50	72.73	0.45
2	57.04	0.14	54.15	0.08
3	54.42	0.09	55.83	0.11
4	94.65	0.89	95.60	0.91
5	87.50	0.75	85.94	0.72
6	76.90	0.54	77.98	0.56
7	72.84	0.46	73.80	0.48
8	93.23	0.86	92.90	0.86
9	85.63	0.71	86.88	0.74
Mean	77.46	0.55	77.31	0.55
Std	14.52	0.29	14.90	0.30

**Table 7:** Performance of inter-subject learning for single-trial decoding.

**ID**	***CA*_*ST*_**			
	**Adam**		**SGDM**	
	**Test** **(%)**	**Kappa** **(Test)**	**Test** **(%)**	**Kappa** **(Test)**
1	73.30	0.47	72.04	0.44
2	57.54	0.15	55.18	0.10
3	55.00	0.10	55.76	0.12
4	86.26	0.73	83.95	0.68
5	66.25	0.33	68.74	0.38
6	74.62	0.49	73.54	0.47
7	71.59	0.43	70.18	0.40
8	76.73	0.53	75.78	0.52
9	77.19	0.54	76.77	0.54
Mean	70.94	0.42	70.22	0.40
Std	9.89	0.20	9.45	0.19

**Figure 6:**
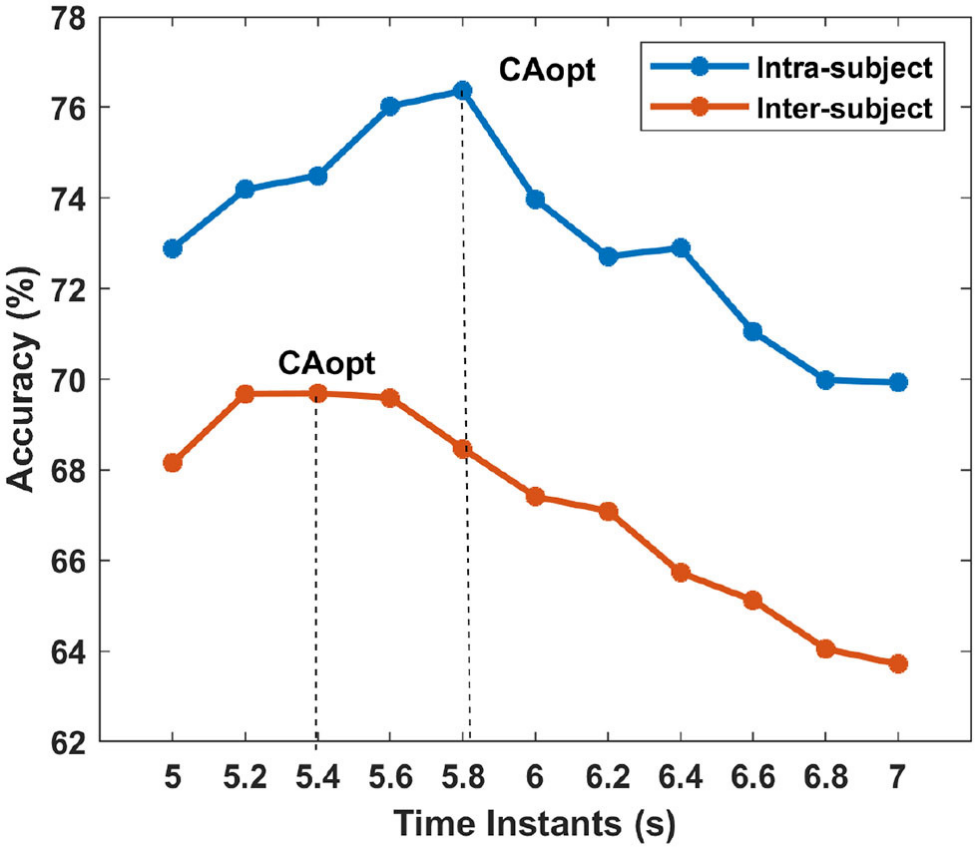
The average accuracy across the subjects for each time instants are shown for intra- and inter-subject learning. The vertical lines show the time-point where is accuracy is maximum for intra- and inter-subject learning, i.e., the average *CA*_*opt*_.

Tables 8, 9 represent the performance of intra- and inter-subject learning accordingly based on *CA*_*opt*_. The classification accuracy of all the participants for all the 11 time instants (5 to 7 s with an interval of 0.2 s) is shown column-wise. The maximum accuracy occurring out of these 11 time instants is the *CA*_*opt*_ for individual participants. For example, in Table 8 the first row represents accuracies achieved for participant 1 for all the 11-time instants out of which the accuracy at 5.8 s was the highest (71.16%). So, the *CA*_*opt*_ for participant 1 is 71.16% observed at 5.8 s. Thus we can see that *CA*_*opt*_ for intra-subject learning was found between 5.2 s and 5.8 s across all the participants, with an average of 76.37% ± 13.91 observed at 5.8 s. A maximum *CA*_*opt*_ of 95.91% was found in participant 4 at 5.6 s, while a minimum *CA*_*opt*_ (55.83%) was found in participant 3 at 5.6 s. Thus, on the basis of *CA*_*opt*_, 7 out of 9 participants performed beyond the BCI literacy threshold. Again, for inter-subject learning the average *CA*_*opt*_ was found to be 69.69% ± 9.23 at 5.4 s, which was significantly (*p* < 0.05, Wilcoxon signed-rank test) lower than average *CA*_*opt*_ for intra-subject learning, although very close to the BCI literacy threshold. The maximum performance was found in participant 4 (*CA*_*opt*_ = 86.80% at 5.6 s), while the minimum performance was found in participant 2 (*CA*_*opt*_ = 56.80% at 5 s). Again, 6 out of 9 participants crossed the BCI performance threshold of 70% in this case. The accuracy of decoding throughout different time instants within the trial is also shown in Figure 6 for intra- and inter-subject learning, which shows that the performance was significantly higher (*p* < 0.05, Wilcoxon signed-rank test) in the case of intra-subject than in inter-subject learning, while the *CA*_*opt*_ occurred earlier in the inter-subject case than in intra-subject. Interestingly, the accuracy curves in both the cases peaked in the middle and gradually reduced at the end of the trial. It is to be noted that the optimum time point of feedback for CNN based inter-subject transfer learning for the dataset used is 5.4 s (i.e., +2.4 s after cue), yielding an average accuracy (*CA*_*opt*_) close to 69.69% (Table 9). This observation is also according to the ERD pattern of the MI-datasets (Tangermann et al., 2012) where the bandpower of sensorimotor rhythm reaches its bottom and stabilizes until the MI is stopped. This indirectly shows the neurophysiological relevance of the features generated by the CNN.

**Table 8:** Performance of intra-subject learning for *CA*_*opt*_ (highlighted in bold for each subject id).

	**Accuracy (%) at different time instants within a trial**										
**ID**	**5s**	**5.2s**	**5.4s**	**5.6s**	**5.8s**	**6s**	**6.2s**	**6.4s**	**6.6s**	**6.8s**	**7s**
1	68.03	67.71	67.40	68.65	**71.16**	68.03	67.71	68.03	68.65	68.03	68.03
2	56.68	55.96	56.32	55.60	**58.48**	53.07	51.62	57.76	51.99	52.71	55.96
3	56.54	**56.18**	56.18	55.83	56.18	55.12	53.36	53.71	51.94	50.88	54.77
4	91.19	93.40	95.28	**95.91**	95.60	94.65	92.14	88.05	88.99	85.22	82.08
5	78.13	79.69	77.50	83.75	**84.06**	82.50	81.56	79.06	79.38	77.81	78.44
6	74.01	**75.09**	73.29	74.01	73.65	71.84	72.20	71.12	70.40	69.68	69.31
7	67.73	69.01	**71.57**	71.57	70.93	64.86	66.77	70.61	64.86	66.77	61.02
8	89.03	92.26	**92.58**	92.58	92.26	91.61	88.71	87.42	84.84	81.29	82.58
9	74.69	78.44	80.31	**86.25**	85.00	84.06	80.31	80.31	78.44	77.50	77.19
Mean	72.89	74.19	74.49	76.02	**76.37**	73.97	72.71	72.90	71.05	69.99	69.93
Std	12.29	13.55	13.82	14.74	13.91	15.12	14.36	12.06	13.27	12.02	10.85

*The bold values represent the maximum value in the respective rows of the table, which means at what time point the maximum accuracy is reached for a particular subject*.

**Table 9:** Performance of inter-subject learning for *CA*_*opt*_ (highlighted in bold for each subject id).

	**Accuracy (%) at different time instants within a trial**										
**ID**	**5s**	**5.2s**	**5.4s**	**5.6s**	**5.8s**	**6s**	**6.2s**	**6.4s**	**6.6s**	**6.8s**	**7s**
1	67.04	**71.21**	69.82	70.51	68.98	67.73	67.45	65.51	66.34	67.59	66.06
2	**56.80**	54.73	55.77	53.11	55.18	54.73	53.85	51.92	54.59	52.37	54.59
3	55.30	56.21	**57.42**	55.45	55.30	53.48	54.09	52.88	52.88	51.52	51.06
4	81.63	84.76	85.31	**86.80**	84.76	83.13	78.91	78.37	75.51	74.42	74.69
5	63.76	66.11	66.11	**66.67**	66.67	65.28	66.11	64.45	61.83	61.13	62.66
6	69.08	70.77	69.08	**72.00**	69.08	68.92	68.46	66.77	67.69	68.62	67.23
7	73.00	**73.98**	72.15	71.17	68.21	66.24	67.37	65.68	64.56	63.99	61.46
8	75.10	**76.60**	75.51	75.78	73.33	73.33	73.61	72.24	69.39	66.39	66.26
9	71.63	72.74	**76.08**	74.83	74.69	73.85	73.99	73.71	73.30	70.38	69.40
Mean	68.15	69.68	**69.69**	69.59	68.47	67.41	67.09	65.73	65.12	64.05	63.71
Std	8.52	9.51	9.23	10.32	9.23	9.26	8.49	8.83	7.69	7.82	7.30

*The bold values represent the maximum value in the respective rows of the table, which means at what time point the maximum accuracy is reached for a particular subject*.

**Figure 7:**
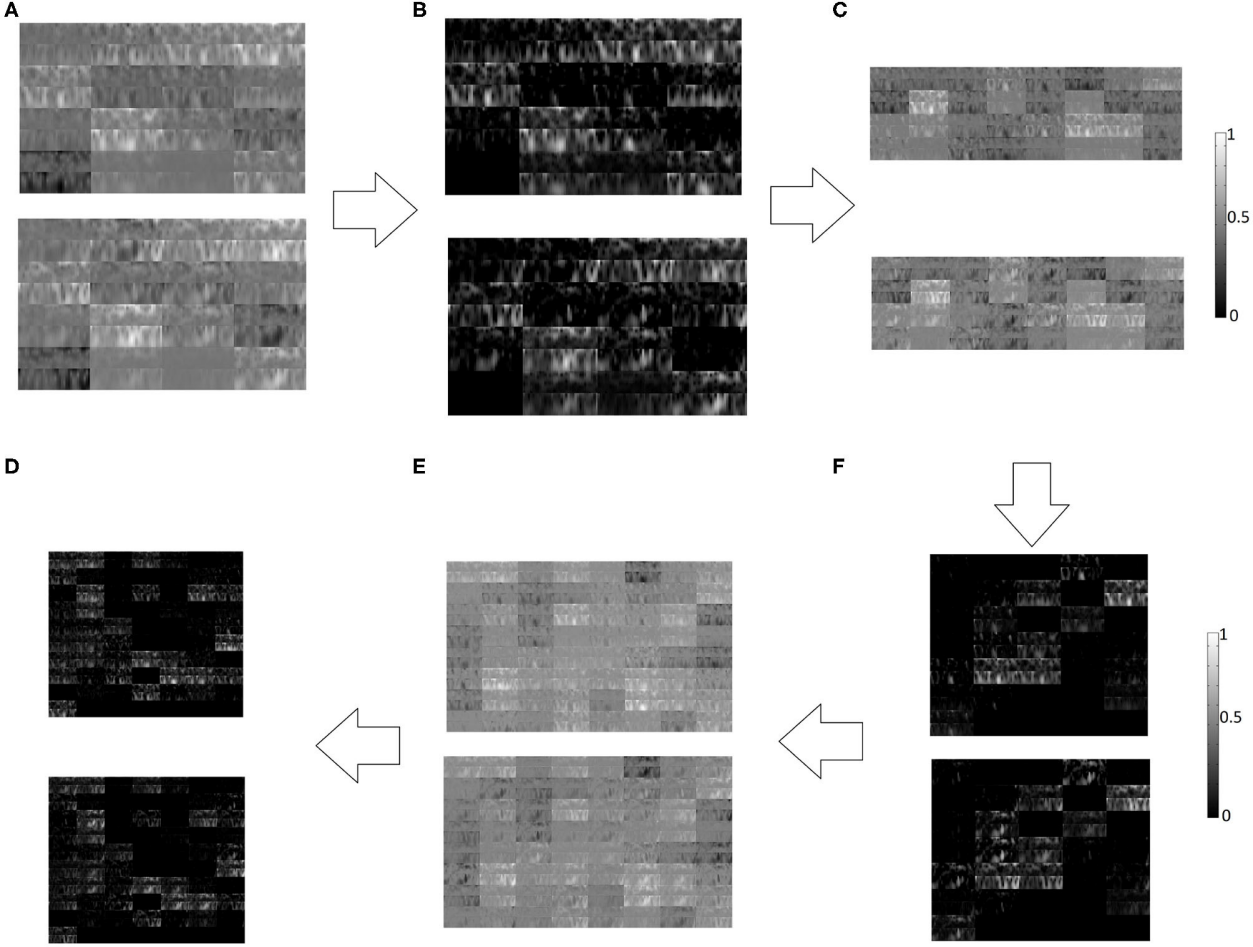
Left hand MI (Top panel) and right hand MI (Bottom panel) features generated at different layers of CNN, **(A)** Convolutional Layer 1, **(B)** ReLU Layer 1, **(C)** Convolutional Layer 2, **(D)** ReLU Layer 2, **(E)** Convolutional Layer 3, **(F)** ReLU Layer 3.

A typical example of features generated at different layers of CNN has been shown in Figure 7. The features for left and right hand MI are shown one on top of the other for successive layers of convolutional and ReLU layers. Although such representations of the activations are not relatable directly with the neurophysiological interpretation due to several transformations on the original image, these are better interpretable by the trained CNN model.

## 4. Discussion

This paper establishes the feasibility of CNN based architectures in inter-subject continuous decoding of MI-related EEG signals while adapting CNN architecture against inter-subject variabilities using a novel concept called Mega Blocks. So far, the issue of inter-subject transfer learning has not been addressed with regards to continuous neurofeedback as the previous studies have mostly concentrated on single-trial classification. Here, we have shown inter-subject transfer learning performance of CNN based architectures for continuous decoding on the standard EEG dataset of BCI Competition-IV using two popular methods: Adam and SGDM. Earlier attempts at classifying MI signals using CNN were limited to intra-subject learning (Tabar and Halici, 2016), while our study deals with inter-subject transfer learning. The significance of designing an inter-subject transfer learning paradigm over intra-subject learning is that we can save the calibration time by making use of the data recorded in previous sessions. Some recent papers have reported inter-subject classification using CNN (Lawhern et al., 2018; Zubarev et al., 2019). Lawhern et al. (2018) in their EEGNet model argued that a single CNN can perform over multiple EEG paradigms such as P300, ERN, MRCP, and SMR, although EEGNet did not perform significantly better than conventional FBCSP approach. Additionally, DeepConvNet (Lawhern et al., 2018) is shown to have performed significantly lower than FBCSP whereas in our case the Mega Block based deep learning architecture (Architectre-2) performed as good as FBCSP (Raza et al., 2016) and further showed validity for inter-subject learning. Moreover, the performance of EEGNet was shown based on cross-validation over the training data, whereas the performance of Architecture-2 is shown on the test data. However, the work in Zubarev et al. (2019) was focused on inter-subject learning in MEG, and showed significantly better performance than other CNN based classification techniques in BCI, although the performance was not reported on EEG. An advantage of the proposed CNN model is that it can be applied for continuous decoding within a trial, while the models in Lawhern et al. (2018) and Zubarev et al. (2019) are shown to have performed well for a single-trial decoding. Most importantly, these studies have not shown how CNN can be used for continuous decoding, an area that is vital for contingent neurofeedback for restorative BCI applications, while the proposed technique provides a complete solution for CNN based MI-BCI combining inter-subject transfer learning with continuous decoding. Another aspect of our model is automatic parameter optimization during training using the implemented Bayesian optimization. The training time (approx. 1,796 s) of Architecture-2 is comparable to a shallowConvNet, although unlike shallowConvNet here the number of trainable parameters doesn't increase with the number of channels used. The average kappa value for intra-subject classification reported by Gandhi et al. (2015) on BCI competition IV 2b dataset was 0.54 and 0.51 on the evaluation set 04E and 05E, respectively although they used a recurrent quantum neural network (RQNN). In our case, the average kappa for intra-subject classification is 0.55 (see Table 6) for both Adam and SGDM. However, we know that the poor outcome in the case of subject 2 and subject 3 is mostly due to the poor quality of the data as evident by the BCI competition results (BCI-Competition, 2008), wherein these two subjects performed worst in all top 6 submissions. Hence, if we remove these two subjects from the calculation then average kappa for intra-subject classification turns out to be 0.67 for both Adam and SGDM, while the same in Gandhi et al. (2015) is 0.55 (excluding subject 2 and 3) for combined evaluation set 04E and 05E. Thus we can see that the performance of CNN for intra-subject learning is far better than RQNN and also the difference is statistically significant (*p* < 0.05, Wilcoxon signrank test). More importantly, it should be noted that our paper is focused on giving an acceptable solution for inter-subject transfer learning in MI task in which case the proposed method gives a satisfactory average kappa value of 0.42 (including all 9 subjects, see Table 7) and 0.50 (ignoring subject 2 and subject 3). It is to be noted that in Gandhi et al. (2015) there was neither evaluation for inter-subject transfer learning performance nor for continuous decoding.

The classification accuracy results highlight an important finding that it is the tuning of the hyperparameters of CNN, which is more effective than the choice of the adaptive training method. This is revealed from the fact that there were no significant (*p* < 0.05) differences between the performance of Adam and SGDM, except in the case of *CA*_*gross*_ in inter-subject learning wherein the average difference in average accuracy is only 0.62%. A probable reason for this can be found from the comments made by Wilson et al. (2017), which states that the choice of the adaptive method (such as Adam and SGDM), makes a difference in optimization-free iterative search procedures (such as GANs and Q-learning). This indicates that as we have used an optimization dependent learning architecture such as CNN, the hyperparameter tuning plays a more vital role in the performance of the classifier.

The inter-subject transfer learning performance was also compared against the intra-subject classification to determine how much compromise is needed in terms of accuracy in order to avoid subject-specific calibration and whether this compromise is worthwhile. The performance of inter-subject transfer learning is found to be significantly lower (*p* < 0.05) than the intra-subject learning both in terms of continuous decoding (*CA*_*gross*_) and single-trial decoding (*CA*_*ST*_) irrespective of the adaptive training methods (Adam or SGDM) used. A possible reason for the lower performance could be the use of a large amount of pooled data from the rest of the 8 participants in the leave-one-out method while some participants (especially participant 2 and 3) had poor quality of data which may have impacted the trained models. However, the average inter-subject transfer learning accuracy for *CA*_*ST*_ was found to be higher than 70%, the BCI performance threshold. Single-trial decoding is sufficient for issuing triggered neurofeedback, which is a widely used paradigm for the rehabilitation of motor functionality (Buch et al., 2008; Ramos-Murguialday et al., 2013; Ono et al., 2014). Thus we can say that the proposed transfer learning architecture can be incorporated into motor rehabilitation paradigms without compromising on an acceptable performance criterion. Another important point to be noted is that the worst-performing subjects (subject 2 and subject 3), and the best performing subject (subject 4) are consistent across intra- or inter-subject learning conditions, which may indicate poor quality of the data and not the strength of the algorithm which negatively affected the average accuracy of inter-subject transfer learning. Hence, if we ignore subject 2 and 3, the performance of inter-subject transfer learning increases further both in terms of *CA*_*gross*_ (Adam: 70.84% and SGDM: 71.49%), and *CA*_*ST*_ (Adam: 75.13% and SGDM: 74.43%).

Previous literature on inter-subject transfer learning using CSP yielded the best average accuracy of 79% on BCI Competition III, dataset IVa, where the number of subjects was 5 (Devlaminck et al., 2011). Tangent space features drawn from the Riemannian geometry framework were used for transfer learning using BCI competition IV, dataset 2a, which achieved an average leave-one-subject-out-cross-validation accuracy of 75.52% (Gaur et al., 2019a). In a recent study, Halme and Parkkonen reported inter-subject transfer learning accuracy in EEG of 67.7% on their own experimental data using CSP with logistic regression (Halme and Parkkonen, 2018). Although a direct comparison is not possible here as the datasets used in Gaur et al. (2019a), Halme and Parkkonen (2018), and Devlaminck et al. (2011) were different but the average of 7 out of 9 subjects [ignoring subject 2 and subject 3 due to poor data quality as revealed by BCI competition results (BCI-Competition, 2008)] in our case achieved an average single-trial classification accuracy close to 75% (Adam: 75.13% and SGDM: 74.43%). It is noteworthy that previous studies on inter-subject transfer learning mentioned above did not deal with continuous decoding and used traditional approaches rather than deep learning. The work also shows that inter-subject transfer learning in MI with CNN based architecture is more sensitive to the tuning of hyperparameters rather than the choice of adaptive training methods as both Adam and SGDM performed equally well in this case.

Potential applications where the obtained results can be useful include primarily the neurorehabilitative BCI systems where continuous and meaningful neurofeedback is essential for motor recovery (Chowdhury et al., 2018a). Apart from that, the asynchronous BCI uses for activities of daily living (ADL) by the completely locked-in patients can also make use of such techniques for controlling assistive robotic devices (Bhattacharyya et al., 2017; Tariq et al., 2018). Another important application could be the telepresence robot control by the motor-disabled patients towardz enhanced independence (Carlson et al., 2013) which needs continuous decoding with minimal calibration overhead.

One of the limitations of this study is that we combined the EEG channels depthwise similar to RGB images which could cause problems in very high dimensional datasets such as in magnetoencephalography (MEG) or very high dimensional EEG recordings. Possible future work to avoid such a problem is to use dimensionality reduction techniques such as ReliefF (RF) or Infinite Latent Feature Selection (ILFS) (Roy et al., 2019b) before input image generation. Also, to increase the number of training examples to feed into the CNN, Generalized Adversarial Networks (GAN) (Goodfellow et al., 2014) could be used rather than the segmentation of trials for creating training examples. Another limitation of using CNN based architectures is that the generated features are not relatable directly with the neurophysiology. Therefore, we need better visualization techniques to enhance the interpretability of activations found in different layers which could have some neurophysiological significance. Other future works may involve making deep learning models more explainable to address the generalizability of inter-subject decoding. Another important challenge is to make them usable for large-scale real-world deployment for complex BCI problems (Zhang et al., 2019).

## 5. Conclusion

This paper presents the feasibility of inter-subject continuous decoding utilizing CNN based deep learning frameworks using a novel concept called Mega Blocks which makes it adaptive against inter-subject variabilities in the EEG data. The study addresses the long-standing issue of making an MI-BCI calibration-free as well as suitable for continuous decoding, which so far has not been addressed using a CNN-based learning approach. This could spawn the next generation of MI-BCI systems, especially in the domain of neurorehabilitation, where reducing the calibration needs and providing continuous feedback play a vital role in enhancing user-experience and thus leverage rehabilitative potential.

## Data Availability Statement

Publicly available datasets were analyzed in this study. This data can be found here: http://www.bbci.de/competition/iv/desc_2b.pdf.

## Author Contributions

SR and AC have conceptualized the idea and finalized the data processing pipeline. SR performed the data analysis and extracted results. AC and SR have written the paper. KM and GP supervised the project and performed the internal review of the paper. All authors contributed to the article and approved the submitted version.

## Conflict of Interest

The authors declare that the research was conducted in the absence of any commercial or financial relationships that could be construed as a potential conflict of interest.

## References

[B1] Arvaneh M. Robertson I. Ward T. E. (2014). “Subject-to-subject adaptation to reduce calibration time in motor imagery-based brain-computer interface,” in 2014 36th Annual International Conference of the IEEE Engineering in Medicine and Biology Society (Chicago, IL), 6501–6504. 10.1109/EMBC.2014.6945117 25571485

[B2] BCI-Competition (2008). BCI Competition 2008–Graz Data Set B. 21374997

[B3] Bhattacharyya S. Konar A. Tibarewala D. N. (2017). Motor imagery and error related potential induced position control of a robotic arm. IEEE/CAA J. Automat. Sin. 4, 639–650. 10.1109/JAS.2017.7510616

[B4] Blankertz B. Vidaurre C. (2009). Towards a cure for BCI illiteracy: machine learning based co-adaptive learning. BMC Neurosci. 10:P85. 10.1186/1471-2202-10-S1-P8519946737

[B5] Buch E. Weber C. Cohen L. G. Braun C. Dimyan M. A. Ard T.. (2008). Think to move: a neuromagnetic brain-computer interface (BCI) system for chronic stroke. Stroke 39, 910–917. 10.1161/STROKEAHA.107.505313 18258825 PMC5494966

[B6] Bundy D. T. Souders L Baranyai K. Leonard L. Schalk G. Coker R.. (2017). Contralesional brain-computer interface control of a powered exoskeleton for motor recovery in chronic stroke survivors. Stroke 48, 1908–1915. 10.1161/STROKEAHA.116.016304 28550098 PMC5482564

[B7] Carlson T. Tonin L. Perdikis S. Leeb R. Millan J. R. (2013). “A hybrid BCI for enhanced control of a telepresence robot,” in 2013 35th Annual International Conference of the IEEE Engineering in Medicine and Biology Society (EMBC) (Osaka), 3097–3100. 10.1109/EMBC.2013.6610196 24110383

[B8] Chowdhury A. Meena Y. K. Raza H. Bhushan B. Uttam A. K. Pandey N.. (2018a). Active physical practice followed by mental practice using BCI-driven hand exoskeleton: a pilot trial for clinical effectiveness and usability. IEEE J. Biomed. Health Inform. 22, 1786–1795. 10.1109/JBHI.2018.2863212 30080152

[B9] Chowdhury A. Raza H. Meena Y. K. Dutta A. Prasad G. (2018b). Online covariate shift detection-based adaptive brain-computer interface to trigger hand exoskeleton feedback for neuro-rehabilitation. IEEE Trans. Cogn. Dev. Syst. 10, 1070–1080. 10.1109/TCDS.2017.2787040

[B10] Chowdhury A. Raza H. Meena Y. K. Dutta A. Prasad G. (2019). An EEG-EMG correlation-based brain-computer interface for hand orthosis supported neuro-rehabilitation. J. Neurosci. Methods 312, 1–11. 10.1016/j.jneumeth.2018.11.010 30452976

[B11] Devlaminck D. Wyns B. Grosse-Wentrup M. Otte G. Santens P. (2011). Multisubject learning for common spatial patterns in motor-imagery BCI. Comput. Intell. Neurosci. 2011, 1–9. 10.1155/2011/217987 22007194 PMC3191786

[B12] Fahimi F. Zhang Z. Goh W. B. Lee T.-S. Ang K. K. Guan C. (2019). Inter-subject transfer learning with an end-to-end deep convolutional neural network for EEG-based BCI. J. Neural Eng. 16:026007. 10.1088/1741-2552/aaf3f6 30524056

[B13] Fazli S. Popescu F. Danoczy M. Blankertz B. Muller K.-R. Grozea C. (2009). Subject-independent mental state classification in single trials. Neural Netw. 22, 1305–1312. 10.1016/j.neunet.2009.06.003 19560898

[B14] Foldes S. T. Weber D. J. Collinger J. L. (2015). MEG-based neurofeedback for hand rehabilitation. J. Neuroeng. Rehabil. 12:85. 10.1186/s12984-015-0076-7 26392353 PMC4578759

[B15] Gandhi V. Prasad G. Coyle D. Behera L. McGinnity T. M. (2015). Evaluating quantum neural network filtered motor imagery brain-computer interface using multiple classification techniques. Neurocomputing 170, 161–167. 10.1016/j.neucom.2014.12.114

[B16] Gaur P. McCreadie K. Pachori R. B. Wang H. Prasad G. (2019a). Tangent space features-based transfer learning classification model for two-class motor imagery brain-computer interface. Int. J. Neural Syst. 29:1950025. 10.1142/S0129065719500254 31711330

[B17] Gaur P. Pachori R. B. Wang H. Prasad G. (2019b). An automatic subject specific intrinsic mode function selection for enhancing two-class EEG-based motor imagery-brain computer interface. IEEE Sens. J. 19, 6938–6947. 10.1109/JSEN.2019.2912790

[B18] Gelbart M. A. Snoek J. Adams R. P. (2014). Bayesian optimization with unknown constraints. arXiv[Preprint].arXiv:1403.5607. 10.5555/3020751.302077831798351

[B19] Goodfellow I. J. Pouget-Abadie J. Mirza M. Xu B. Warde-Farley D. Ozair S. (2014). “Generative adversarial nets,” in Proceedings of the 27th International Conference on Neural Information Processing Systems, Vol. 2 (Cambridge, MA: MIT Press), 2672–2680.

[B20] Halme H. Parkkonen L. (2018). Across-subject offline decoding of motor imagery from MEG and EEG. Sci. Rep. 8:10087 10.1038/s41598-018-28295-z 29973645 PMC6031658

[B21] He K. Zhang X. Ren S. Sun J. (2016). “Deep residual learning for image recognition,” in 2016 IEEE Conference on Computer Vision and Pattern Recognition (CVPR), 770–778.

[B22] Jayaram V. Alamgir M. Altun Y. Scholkopf B. Grosse-Wentrup M. (2016). Transfer learning in brain-computer interfaces. IEEE Comput. Intell. Mag. 11, 20–31. 10.1109/MCI.2015.2501545

[B23] Jiao Y. Zhang Y. Chen X. Yin E. Jin J. Wang X.. (2019). Sparse group representation model for motor imagery EEG classification. IEEE J. Biomed. Health Inform. 23, 631–641. 10.1109/JBHI.2018.2832538 29994055

[B24] Jin J. Sellers E. W. Zhang Y. Daly I. Wang X. Cichocki A. (2013). Whether generic model works for rapid ERP-based BCI calibration. J. Neurosci. Methods 212, 94–99. 10.1016/j.jneumeth.2012.09.020 23032116 PMC3658461

[B25] Jin Z. Gao D. Zhou G. Zhang Y. (2020). EEG classification using sparse Bayesian extreme learning machine for brain-computer interface. Neural Comput. Appl. 32, 6601–6609. 10.1007/s00521-018-3735-3

[B26] Johnson R. Zhang T. (2013). “Accelerating stochastic gradient descent using predictive variance reduction,” in Proceedings of the 26th International Conference on Neural Information Processing Systems, NIPS'13 (Lake Tahoe: Curran Associates Inc.), 315–323.

[B27] Kang H. Nam Y. Choi S. (2009). Composite common spatial pattern for subject-to-subject transfer. IEEE Signal Process. Lett. 16, 683–686. 10.1109/LSP.2009.2022557

[B28] Kindermans P.-J. Tangermann M. Muller K.-R. Schrauwen B. (2014). Integrating dynamic stopping, transfer learning and language models in an adaptive zero-training ERP speller. J. Neural Eng. 11:035005. 10.1088/1741-2560/11/3/035005 24834896

[B29] Kingma D. P. Ba J. (2014). ADAM: a method for stochastic optimization. arXiv[Preprint].arXiv:1412.6980.

[B30] Krizhevsky A. Sutskever I. Hinton G. E. (2012). “Imagenet classification with deep convolutional neural networks,” in Advances in Neural Information Processing Systems 25, eds F. Pereira, C. J. C. Burges, L. Bottou, and K. Q. Weinberger (Lake Tahoe: Curran Associates, Inc.), 1097–1105.

[B31] Kwon O. Lee M. Guan C. Lee S. (2019). Subject-independent brain-computer interfaces based on deep convolutional neural networks. IEEE Trans. Neural Netw. Learn. Systems. 10.1109/TNNLS.2019.2946869. [Epub ahead of print]. 31725394

[B32] Lawhern V. J. Solon A. J. Waytowich N. R. Gordon S. M. Hung C. P. Lance B. J. (2018). EEGNet: a compact convolutional neural network for EEG-based brain-computer interfaces. J. Neural Eng. 15:056013. 10.1088/1741-2552/aace8c 29932424

[B33] Li Y. Kambara H. Koike Y. Sugiyama M. (2010). Application of covariate shift adaptation techniques in brain-computer interfaces. IEEE Trans. Biomed. Eng. 57, 1318–1324. 10.1109/TBME.2009.2039997 20172795

[B34] Lotte F. Bougrain L. Cichocki A. Clerc M. Congedo M. Rakotomamonjy A.. (2018). A review of classification algorithms for EEG-based brain-computer interfaces: a 10 year update. J. Neural Eng. 15:031005. 10.1088/1741-2552/aab2f2 29488902

[B35] Lotte F. Guan C. (2011). Regularizing common spatial patterns to improve BCI designs: unified theory and new algorithms. IEEE Trans. Biomed. Eng. 58, 355–362. 10.1109/TBME.2010.2082539 20889426

[B36] Lu N. Li T. Ren X. Miao H. (2017). A deep learning scheme for motor imagery classification based on restricted Boltzmann machines. IEEE Trans. Neural Syst. Rehabil. Eng. 25, 566–576. 10.1109/TNSRE.2016.2601240 27542114

[B37] Morone G. Pisotta I. Pichiorri F. Kleih S. Paolucci S. Molinari M.. (2015). Proof of principle of a brain-computer interface approach to support poststroke arm rehabilitation in hospitalized patients: design, acceptability, and usability. Arch. Phys. Med. Rehabil. 96, S71–S78. 10.1016/j.apmr.2014.05.026 25721550

[B38] Ono T. Shindo K. Kawashima K. Ota N. Ito M. Ota T.. (2014). Brain-computer interface with somatosensory feedback improves functional recovery from severe hemiplegia due to chronic stroke. Front. Neuroeng. 7:19. 10.3389/fneng.2014.00019 25071543 PMC4083225

[B39] Pfurtscheller G. da Silva F. L. (1999). Event-related EEG/MEG synchronization and desynchronization: basic principles. Clin. Neurophysiol. 110, 1842–1857. 10.1016/S1388-2457(99)00141-8 10576479

[B40] Pohlmeyer E. A. Mahmoudi B. Geng S. Prins N. W. Sanchez J. C. (2014). Using reinforcement learning to provide stable brain-machine interface control despite neural input reorganization. PLoS ONE 9:e87253. 10.1371/journal.pone.0087253 24498055 PMC3907465

[B41] Prins N. W. Sanchez J. C. Prasad A. (2017). Feedback for reinforcement learning based brain-machine interfaces using confidence metrics. J. Neural Eng. 14:036016. 10.1088/1741-2552/aa6317 28240598

[B42] Qian N. (1999). On the momentum term in gradient descent learning algorithms. Neural Netw. 12, 145–151. 10.1016/S0893-6080(98)00116-6 12662723

[B43] Ramos-Murguialday A. Broetz D. Rea M. Laer L. Yilmaz Z. Brasil F. L.. (2013). Brain-machine interface in chronic stroke rehabilitation: a controlled study. Ann. Neurol. 74, 100–108. 10.1002/ana.23879 23494615 PMC3700597

[B44] Raza H. Cecotti H. Li Y. Prasad G. (2016). Adaptive learning with covariate shift-detection for motor imagery-based brain-computer interface. Soft Comput. 20, 3085–3096. 10.1007/s00500-015-1937-5

[B45] Raza H. Rathee D. Zhou S.-M. Cecotti H. Prasad G. (2019). Covariate shift estimation based adaptive ensemble learning for handling non-stationarity in motor imagery related EEG-based brain-computer interface. Neurocomputing 343, 154–166. 10.1016/j.neucom.2018.04.087 32226230 PMC7086459

[B46] Roy S. McCreadie K. Prasad G. (2019a). “Can a single model deep learning approach enhance classification accuracy of an EEG-based brain-computer interface?” in 2019 IEEE International Conference on Systems, Man and Cybernetics (SMC) (Bari), 1317–1321. 10.1109/SMC.2019.8914623

[B47] Roy S. Rathee D. McCreadie K. Prasad G. (2019b). “Channel selection improves meg-based brain-computer interface,” in 2019 9th International IEEE/EMBS Conference on Neural Engineering (NER) (San Francisco, CA), 295–298. 10.1109/NER.2019.8716948

[B48] Roy Y. Banville H. Albuquerque I. Gramfort A. Falk T. H. Faubert J. (2019). Deep learning-based electroencephalography analysis: a systematic review. J. Neural Eng. 16:051001. 10.1088/1741-2552/ab260c 31151119

[B49] Saha S. Ahmed K. I. U. Mostafa R. Hadjileontiadis L. Khandoker A. (2018). Evidence of variabilities in EEG dynamics during motor imagery-based multiclass brain-computer interface. IEEE Trans. Neural Syst. Rehabil. Eng. 26, 371–382. 10.1109/TNSRE.2017.2778178 29432108

[B50] Saha S. Baumert M. (2020). Intra- and inter-subject variability in EEG-based sensorimotor brain computer interface: a review. Front. Comput. Neurosci. 13:87. 10.3389/fncom.2019.00087 32038208 PMC6985367

[B51] Saha S. Hossain M. S. Ahmed K. Mostafa R. Hadjileontiadis L. Khandoker A.. (2019). Wavelet entropy-based inter-subject associative cortical source localization for sensorimotor BCI. Front. Neuroinform. 13:47. 10.3389/fninf.2019.00047 31396068 PMC6664070

[B52] Schirrmeister R. T. Springenberg J. T. Fiederer L. D. J. Glasstetter M. Eggensperger K. Tangermann M.. (2017). Deep learning with convolutional neural networks for EEG decoding and visualization. Hum. Brain Mapp. 38, 5391–5420. 10.1002/hbm.23730 28782865 PMC5655781

[B53] Seghier M. L. Price C. J. (2018). Interpreting and utilising intersubject variability in brain function. Trends Cogn. Sci. 22, 517–530. 10.1016/j.tics.2018.03.003 29609894 PMC5962820

[B54] Spüler M. Bensch M. Kleih S. Rosenstiel W. Bogdan M. Kübler A. (2012). Online use of error-related potentials in healthy users and people with severe motor impairment increases performance of a p300-bci. Clin. Neurophysiol. 123, 1328–1337. 10.1016/j.clinph.2011.11.082 22244309

[B55] Sussillo D. Stavisky S. D. Kao J. C. Ryu S. I. Shenoy K. V. (2016). Making brain-machine interfaces robust to future neural variability. Nat. Commun. 7:13749. 10.1038/ncomms13749 27958268 PMC5159828

[B56] Tabar Y. R. Halici U. (2016). A novel deep learning approach for classification of EEG motor imagery signals. J. Neural Eng. 14:016003. 10.1088/1741-2560/14/1/016003 27900952

[B57] Tangermann M. Muller K.-R. Aertsen A. Birbaumer N. Braun C. Brunner C.. (2012). Review of the BCI competition IV. Front. Neurosci. 6:55. 10.3389/fnins.2012.00055 22811657 PMC3396284

[B58] Tariq M. Trivailo P. M. Simic M. (2018). EEG-based BCI control schemes for lower-limb assistive-robots. Front. Hum. Neurosci. 12:312. 10.3389/fnhum.2018.00312 30127730 PMC6088276

[B59] Tu W. Sun S. (2012). A subject transfer framework for EEG classification. Neurocomputing 82, 109–116. 10.1016/j.neucom.2011.10.024

[B60] Vidaurre C. Kawanabe M. von Bunau P. Blankertz B. Muller K. R. (2011). Toward unsupervised adaptation of LDA for brain-computer interfaces. IEEE Trans. Biomed. Eng. 58, 587–597. 10.1109/TBME.2010.2093133 21095857

[B61] Wang P. Lu J. Zhang B. Tang Z. (2015). “A review on transfer learning for brain-computer interface classification,” in 2015 5th International Conference on Information Science and Technology (ICIST) (Changsha), 315–322. 10.1109/ICIST.2015.7288989

[B62] Wilson A. C. Roelofs R. Stern M. Srebro N. Recht B. (2017). “The marginal value of adaptive gradient methods in machine learning,” in Advances in Neural Information Processing Systems 30, eds I. Guyon, U. V. Luxburg, S. Bengio, H. Wallach, R. Fergus, S. Vishwanathan, and R. Garnett (Long Beach, CA: Curran Associates, Inc), 4148–4158.

[B63] Wronkiewicz M. Larson E. Lee A. K. C. (2015). Leveraging anatomical information to improve transfer learning in brain-computer interfaces. J. Neural Eng. 12:046027. 10.1088/1741-2560/12/4/046027 26169961 PMC4527978

[B64] Zanini P. Congedo M. Jutten C. Said S. Berthoumieu Y. (2018). Transfer learning: a Riemannian geometry framework with applications to brain-computer interfaces. IEEE Trans. Biomed. Eng. 65, 1107–1116. 10.1109/TBME.2017.2742541 28841546

[B65] Zhang X. Yao L. Wang X. Monaghan J. Mcalpine D. Zhang Y. (2019). A survey on deep learning based brain computer interface: recent advances and new Frontiers. arXiv. arXiv:1905.04149. 10.1088/1741-2552/abc902 33171452

[B66] Zubarev I. Zetter R. Halme H. Parkkonen L. (2018). Robust and highly adaptable brain-computer interface with convolutional net architecture based on a generative model of neuromagnetic measurements. arXiv[Preprint].arXiv:1805.10981.

[B67] Zubarev I. Zetter R. Halme H.-L. Parkkonen L. (2019). Adaptive neural network classifier for decoding MEG signals. Neuroimage 197, 425–434. 10.1016/j.neuroimage.2019.04.068 31059799 PMC6609925

